# Influence of organizational factors on the offer and success rate of a trial of labor after cesarean section in Belgium: an ecological study

**DOI:** 10.1186/s12884-023-05984-w

**Published:** 2023-09-22

**Authors:** Griet Vandenberghe, An Vercoutere, Nadège Cuvellier, Elke Van Oost, Charlotte Leroy, Régine Goemaes, Monika Laubach, Michel Boulvain, Caroline Daelemans, the B.OSS collaborating group, J. Ackermans, D. Anton, M. Bafort, A. Batter, J. Belhomme, A. Beliard, B. Bollen, V. Boon, J. Bosteels, V. Bracke, G. Ceysens, F. Chaban, F. Chantraine, E. Christiaensen, L. Clabout, P. Cryns, M.-C. Dallequin, B. De Keersmaecker, J. De Keyser, A. De Knijf, P. Scheir, J. De Loose, A. De Vits, T. De Vos, B. Debecker, C. Delforge, J. Deloor, V. Depauw, A. Depierreux, K. Devolder, L. Claes, S. Dirx, C. Eerdekens, P. Emonts, E. Goenen, P. Grandjean, S. Hollemaert, S. Houben, E. Jankelevitch, G. Janssen, J. Quitnelier, Y. Kacem, C. Klay, A. Laurent, J.-F. Legrève, A. Lestrade, C. Lietaer, A. Loccufier, H. Logghe, F. Loumaye, V. Mariman, N. Minten, D. Mortier, K. Mulders, G. Palgen, T. Pezin, K. Polisiou, C. Riera, M. Romain, B. Rombaut, M. Ruymbeke, K. Scharpé, C. Schockaert, A. Segers, E. Serkei, P. Steenhaut, A. Steylemans, B. Thaler, W. Van Dalen, E. Van De Poel, E. Van Deynse, R. Van Dijck, C. Van Holsbeke, L. Van Hoorick, G. Van Olmen, P. Vanballaer, K. Vancalsteren, S. Vandeginste, S. Vandepitte, K. Verbeken, A. Vereecke, M. Verheecke, L. Watkins-Masters, V. Wijckmans, K. Wuyts

**Affiliations:** 1 Department of Obstetrics, Ghent University Hospital, Vrouwenkliniek, Corneel Heymanslaan 10, 9000 Ghent, Belgium; 2 Department of Gynaecology and Obstetrics, Université Libre de Bruxelles (ULB) Hôpital Universitaire de Bruxelles (H.U.B.), Hôpital Erasme, Route de Lennik 808, Brussels, 1070 Belgium; 3 Present Address: Department of Gynaecology and Obstetrics, Centre Hospitalier EpiCURA Site Ath, Ath, Belgium; 4 Present Address: Department of Obstetrics, AZ Maria Halle, Halle, Belgium; 5 Centre d’Epidémiologie Périnatale (CEpiP) Clos Chapelle-Aux-Champs, 30 Bte, B1.30.04 1200 Brussels, Belgium; 6 Study Centre for Perinatal Epidemiology (SPE), Koning Albert II-Laan 35 Bus 29, 1030 Brussels, Belgium; 7 Service of Obstetrics and Prenatal Medecine, Universitair Ziekenhuis Brussel, Laarbeklaan 101, 1090 Brussels, Belgium; 8 Present Address: Obstetrics Division, Department of Woman, Child and Adolescent Medecine, Geneva University Hospitals, Boulevard de la Cluse, 30, 1205 Geneva, Switzerland; 9 The Belgian Obstetrics Surveillance System, Ghent University Hospital, Ghent, Belgium

**Keywords:** Trial of labor after cesarean, Vaginal birth after cesarean, Decision-to-delivery interval, On-call schedule, Emergency cesarean section

## Abstract

**Background:**

Trial of Labor After Cesarean is an important strategy for reducing the overall rate of cesarean delivery. Offering the option of vaginal delivery to a woman with a history of cesarean section requires the ability to manage a potential uterine rupture quickly and effectively. This requires infrastructure and organization of the maternity unit so that the decision-to-delivery interval is as short as possible when uterine rupture is suspected. We hypothesize that the organizational characteristics of maternity units in Belgium have an impact on their proposal and success rates of trial of labour after cesarean section.

**Methods:**

We collected data on the organizational characteristics of Belgian maternity units using an online questionnaire. Data on the frequency of cesarean section, trial of labor and vaginal birth after cesarean section were obtained from regional perinatal registries. We analyzed the determinants of the proposal and success of trial of labor after cesarean section and report the associations as mean proportions.

**Results:**

Of the 101 maternity units contacted, 97 responded to the questionnaire and data from 95 was included in the analysis. Continuous on-site presence of a gynecologist and an anesthetist was associated with a higher proportion of trial of labor after cesarean section, compared to units where staff was on-call from home (51% versus 46%, *p* = 0.04). There is a non-significant trend towards more trial of labor after cesarean section in units with an operating room in or near the delivery unit and a shorter transfer time, in larger units (> 1500 deliveries/year) and in units with a neonatal intensive care unit. The proposal of trial of labor after cesarean section and its success was negatively correlated to the number of cesarean section in the maternity unit (Spearman’ rho = 0.50 and 0.42, *p* value < 0.001).

**Conclusions:**

Organizational differences in maternity units appear to affect the proposal of trial of labor after cesarean section. Addressing these organizational factors may not be sufficient to change practice, given that general tendency to perform a cesarean section in the maternity unit is the main contributor to the percentage of trial of labor after cesarean.

**Supplementary Information:**

The online version contains supplementary material available at 10.1186/s12884-023-05984-w.

## Background

The projections for cesarean section (CS) rates globally are worrisome. It is estimated that 28.5% of women will give birth by CS by 2030 worldwide [1]. In Europe, overall CS rates have remained stable between 2015 and 2019, but vary considerably between countries (16.4% in Finland and 53.1% in Cyprus) [2]. Overuse of CS has been shown to have no additional benefit in reducing maternal or neonatal risks, and increases the costs and the risks to future fertility, pregnancies and health of the children [3, 4]. In Belgium, the CS rate is increasing, with regional rates in 2021 of 22.4%, 20.1% and 22.1% in Wallonia, Brussels and Flanders, respectively [5–7]. The proportion of women with a history of CS was 68.9%, 70.9% and 70.2% of all multiparous women who had a CS in 2021 in Wallonia, Brussels and Flanders, respectively [5–7]. There is a considerable variation in CS rates between maternity units, ranging from 14.2% to 35.3% in 2021 [5–7].

Avoiding the first CS would have the greatest impact on limiting the increase in the CS rate. Another important strategy is to offer women with a history of CS the opportunity to attempt a Trial of Labor After Cesarean (TOLAC) [8, 9]. TOLAC, compared to Elective Repeat Cesarean Section (ERCS), reduces the risks associated with CS in the index and future pregnancies, but carries the risk of uterine rupture, with the associated severe morbidity and mortality [10]. Therefore, TOLAC implies the need for rapid management in case of a suspected uterine rupture. In addition, a CS performed during labor because of failed TOLAC, exposes the mother to more complications than a planned ERCS. When counseling women with a history of CS, obstetricians will weigh the benefits of a successful VBAC against the risks of a failed TOLAC or uterine rupture. The organizational characteristics of the labor and delivery unit are determinants of the speed and safety with which emergency CS can be performed, and can play an important role in the informed decision making process regarding mode of delivery and management during labor in women with a history of CS [11–15].

The estimated TOLAC rate in Belgium (47%) is low compared to other European countries [16]. In Belgium, maternity units are relatively small (ranging from 130 to 3400 deliveries in 2015–2017) [5–7]. We hypothesize that organizational characteristics, including the proximity to the operating room (OR) and the continuous on-site availability of medical staff, may have an impact on the offer of TOLAC and the management of a woman attempting TOLAC in the maternity unit.

## Methods

### Aim

The objective of this study was to assess the impact of organizational determinants on the provision and success of TOLAC in maternity units in Belgium.

### Study design

We performed an ecological study, using data from 2015 to 2017.

### Belgian setting

Belgium is divideded in three different regions from north to south: Flanders, Brussels and Wallonia. Perinatal data are analyzed at the regional level by the Centre for Perinatal Epidemiology for Flanders (Studiecentrum voor Perinatale Epidemiologie, SPE) and for Brussels and Wallonia (Centre d’Epidémiologie Périnatale, CEpiP). SPE and CEpiP collect data on a mandatory basis, covering almost 100% of births in Belgian maternity units and home births. A selected set of perinatal data is recorded by the obstetrician, midwife and neonatologist immediately after birth.

### Study data

An online questionnaire using Research Electronic Data Capture (REDCap) at Ghent University Hospital and Brussels University Hospital was distributed to a contact person in each maternity unit [17, 18]. This questionnaire collected information on the availability of a gynecologist, anesthesiologist and pediatrician on-site 24/7, the presence of a neonatal intensive care unit (NICU) in the hospital, the location of the OR and the estimated transfer time from the delivery room to the OR in the event of an emergency CS. The answers to the questionnaire were linked to the data from each maternity unit obtained from the national perinatal databases, including total number of deliveries, total number of CS, number of ERCS, estimated number of TOLAC based on the number of successful vaginal births (VBAC), and number of unplanned repeat CS. Because the planned mode of delivery is not recorded in perinatal registries, the number of TOLAC was calculated as the sum of the number of VBAC and the number of unplanned repeat CS.

### Statistical analysis

The unit of analysis was the maternity and the proportions of TOLAC and VBAC were treated as continuous variables, without weighting for the number of deliveries. We calculated a mean proportion of TOLAC for each category of the organizational variables. The statistical significance of the differences was assessed using a T-test, and by an ANOVA when there were more than two categories. We assessed the correlations between the proportions of TOLAC and VBAC in case of TOLAC, and the overall CS rate and the proportions of TOLAC and of VBAC using a Spearman correlation coefficient. We performed a multivariable linear regression analysis to evaluate the independent contribution of predictors to the proportion of TOLAC in the unit.

## Results

A total of 101 maternity units were contacted and 97 completed the questionnaire. Two maternity units were excluded from the analysis because of missing data, one in the questionnaire and one in the obstetric report. Ninety-five maternity units were included in the analysis.

There were important organizational differences between larger maternity units with more than 1500 deliveries/year (*n* = 22) compared to smaller units (1500 or less deliveries/year) (*n* = 73), shown in Table 1. A greater proportion of large maternity units had the multidisciplinary medical staff on-site 24/7, a NICU, an OR in the delivery room or on the same floor and reported a shorter transfer time in case of emergency CS, compared with smaller units.

**Table 1:** Location and organizational characteristics, according to the annual number of deliveries in the maternity

	Maternity units with more than 1500 deliveries per year (*n* = 22)	Maternity units with 1500 deliveries or less per year (*n* = 73)	*P* value*
Region			1.0
Flanders	13 (59%)	45 (62%)	
Brussels-Wallonia	9 (41%)	28 (38%)	
Medical staff on site 24/7			
Gynecologist	13 (59%)	11 (15%)	< 0.001
Anesthetist	19 (86%)	33 (45%)	0.001
Pediatrician	12 (55%)	15 (20%)	0.003
Gynecologist and Anesthetist	12 (55%)	8 (11%)	< 0.001
Presence of a NICU	13 (59%)	6 (8%)	< 0.001
Location of the operating room (OR)			< 0.001
In the delivery room	11 (50%)	13 (18%)	
On the same floor	7 (32%)	14 (19%)	
On a different floor	4 (18%)	46 (63%)	
Estimated transfer time to the OR			0.03
1 min or less	12 (55%)	19 (26%)	
2 to 5 min	8 (36%)	49 (67%)	
More than 5 min	2 (9%)	5 (7%)	

^*^ Fisher exact test

There was a small and not statistically significant difference in the overall percentage of CS performed between larger and smaller units (20.4% and 21.2%, respectively, p = 0.31). There was no difference according by unit size in the percentage of women with a history of CS (11%).

The estimated proportion of women who were offered a TOLAC was significantly higher in maternity units in the southern part of the country, compared to Flanders (*p* = 0.02), as shown in Table 2. The presence of a gynecologist and an anesthetist on site was associated with a higher proportion of TOLAC, compared to maternity units where both medical staff are on call from home (*p* = 0.04) (Table 2). The mean proportion of TOLAC is slightly higher when the maternity unit is larger, has a NICU, has an OR in or near the delivery unit and when the estimated transfer time is shorter, but these differences were not statistically significant (Table 2). The organizational variables were not associated with the overall proportion of CS in the maternity unit, or with the success of the TOLAC attempt (Additional Tables 1 and 2).

**Table 2:** Proportion of attempted TOLAC, according to location, size and organization of the maternity

Characteristic (number of maternities)	Proportion of TOLAC	
	Mean (SD)	Range and *p*-value
Location		*p* = 0.02
Flanders (58)	45.2% (7.0)	27% to 61%
Brussels-Wallonia (37)	49.6% (10.0)	31% to 80%
Number of deliveries/years		*p* = 0.27
> 1500 (22)	48.8% (9.1)	37% to 71%
≤ 1500 (73)	46.4% (8.4)	27% to 80%
Gynecologist on-site 24/7		*p* = 0.02
Yes (24)	50.6% (8.7)	37% to 71%
No (71)	45.7% (8.2)	27% to 80%
Anesthetist on-site 24/7		*p* = 0.28
Yes (52)	47.8% (8.4)	33% to 71%
No (43)	45.9% (8.7)	27% to 80%
Pediatrician on-site 24/7		*p* = 0.24
Yes (27)	48.7% (8.9)	37% to 71%
No (68)	46.3% (8.9)	37% to 71%
Both gynecologist & anesthetist on-site		*p* = 0.04
Yes (20)	50.7% (9.1)	37% to 71%
No (75)	45.9% (8.2)	27% to 80%
Presence of NICU		*p* = 0.10
Yes (19)	50.1% (9.3)	37% to 71%
No (76)	46.2% (8.2)	27% to 80%
Location of the operating room (OR)		*p* = 0.48
In the delivery room (24)	48.6% (7.5)	37% to 62%
On the same floor (21)	45.6% (9.9)	31% to 71%
On a different floor (50)	46.7% (8.5)	27% to 80%
Reported transfer time to the OR		*p* = 0.17
1 min or less (31)	47.2% (7.0)	33% to 62%
2 to 5 min (57)	47.7% (9.1)	27% to 80%
More than 5 min (7)	40.1% (7.9)	31% to 52.5%

**Fig. 1:**
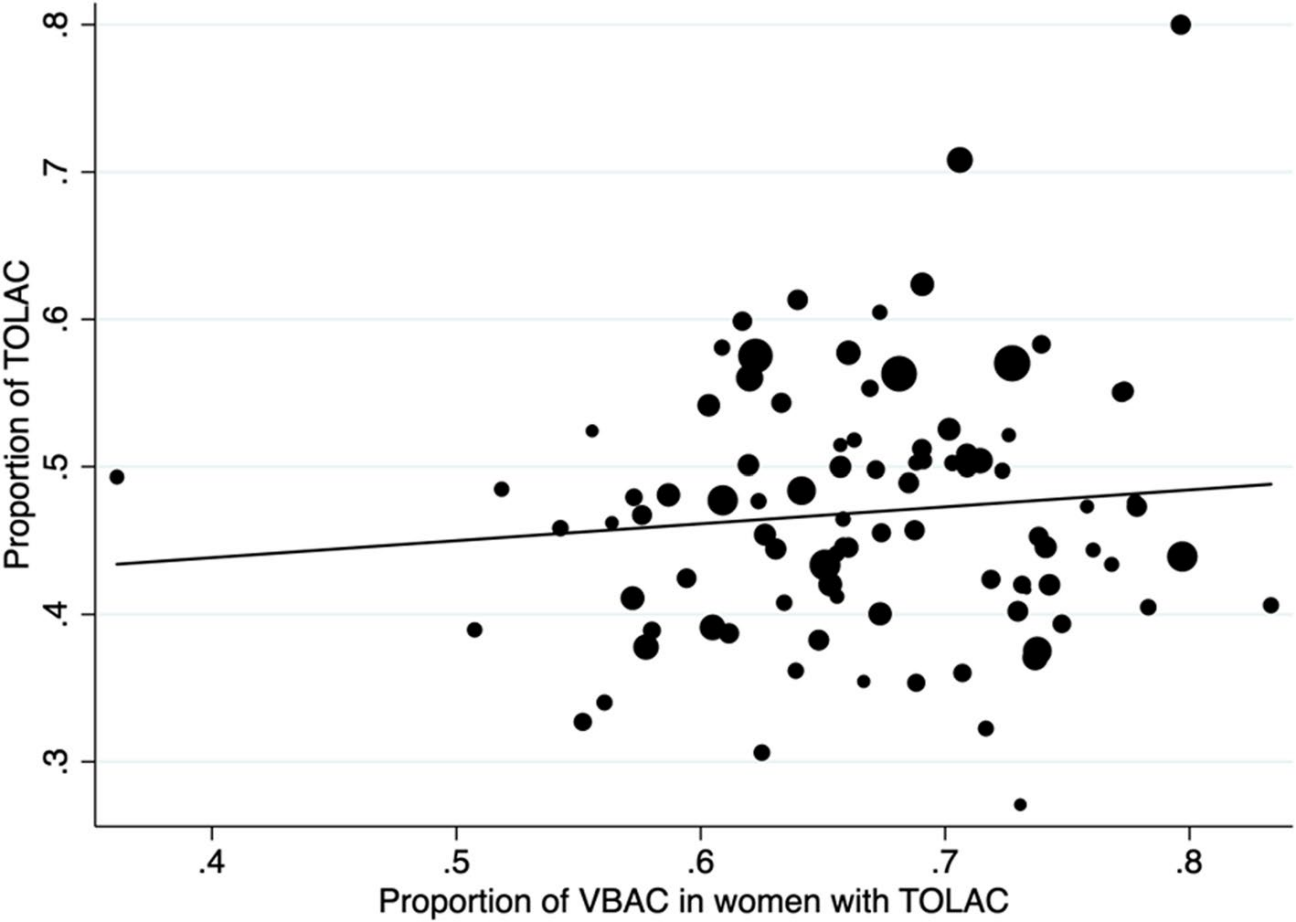
Correlation between the proportion of TOLAC and the proportion of VBAC in case of TOLAC. The size of the dots is weighted by the number of women with history of CS in the maternity unit

**Fig. 2:**
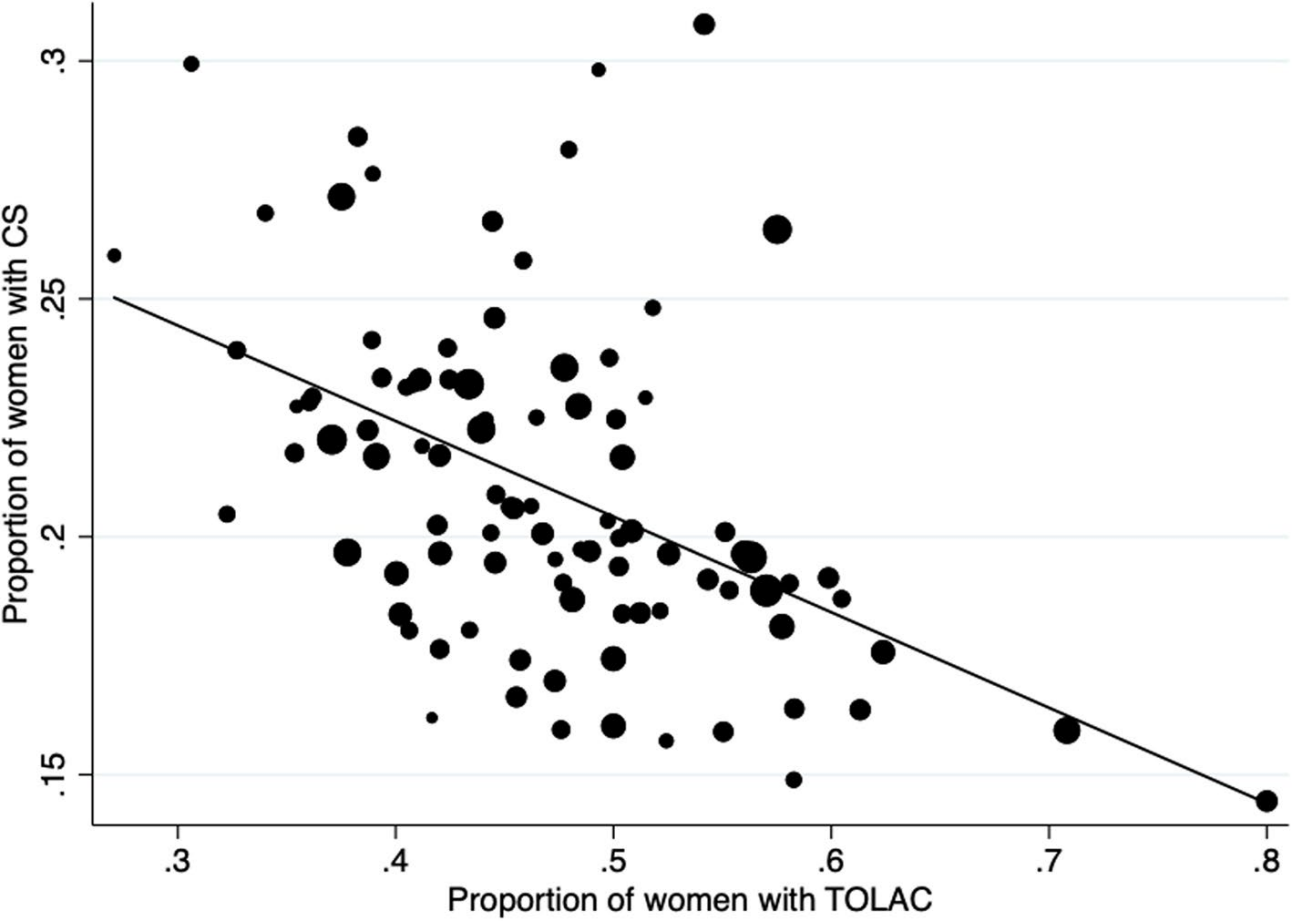
Correlation between the overall proportion of cesarean section and the proportion of TOLAC. The size of the dots is weighted by the number of deliveries per year in the maternity unit

**Fig. 3:**
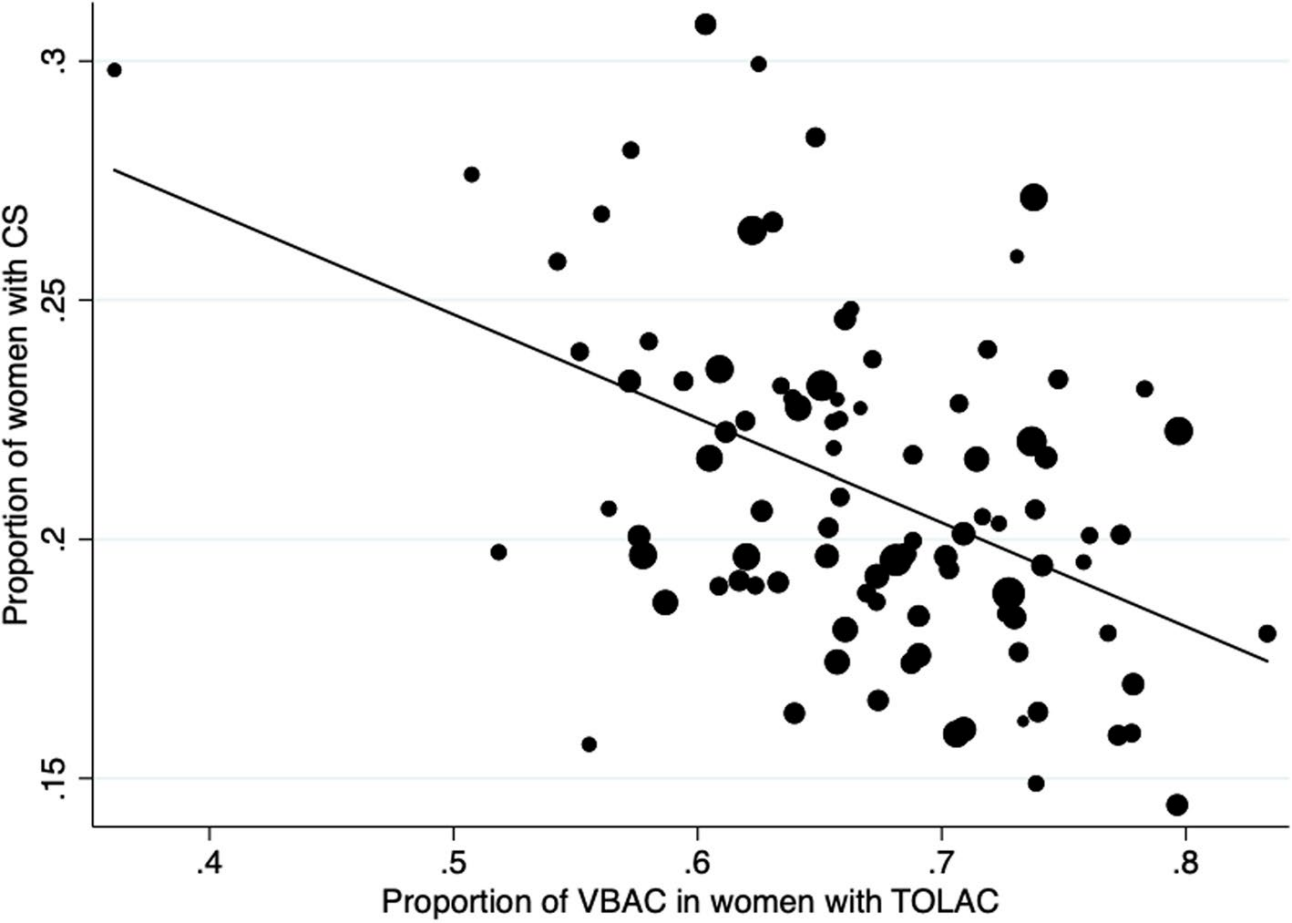
Correlation between the overall proportion of cesarean section and the proportion of VBAC in case of TOLAC. The size of the dots is weighted by the number of deliveries per year in the maternity unit

There was no correlation between the proportion of TOLAC and the overall success rate of TOLAC (Spearman’ rho = 0.06, *p* value = 0.57) (Fig. 1). However, there was a significant negative correlation between the overall proportion of CS in the unit and both the estimated offer of TOLAC (Spearman’ rho = 0.50, *p* value < 0.001) and its success (Spearman’ rho = 0.42, *p* value < 0.001) (Figs. 2 and 3).

Included in a linear multiple regression model, the variables associated in the univariable analysis showed that each percentage increase in the overall proportion of CS was associated with a 1.2% decrease in TOLAC (95%CI: 0.8 to 1.6%, *p* < 0.001), adjusted for the location of the unit and for the presence on-site of a gynecologist and an anesthesiologist on site. The region of the unit was associated with a 4.0% (95%CI: 0.9 to 7.0%, *p* = 0.01) difference in the rate of TOLAC. On-site medical staff presence 24/7 was associated with a 4.2% increase in TOLAC (95%CI: 0.6 to 7.0%, *p* = 0.02), compared with units where medical staff were on-call from home. There was no change in the estimates with adjustment, compared to the results in the univariable analysis, showing that the included variables are independent predictors of the proportion of TOLAC. Adding other organizational variables to the model did not add information because of important associations between maternity units characteristics.

## Discussion

We found important differences between maternity units in the percentage of women with a history of CS who attempted a vaginal delivery. These differences were related to organizational factors, but also to the general tendency of the hospital to perform a CS.

Limitations of our study include the fact that the questionnaire was short to avoid overwhelming busy clinicians. In some units, the on-site gynecologist is a trainee who must wait for a senior colleague to perform a CS. We did not ask for this level of detail. In many maternity units in Belgium the CS room is located outside the delivery room, even on a different floor, especially in smaller hospitals. Nevertheless, most transfer times from the delivery room to the OR are reported to be less than five minutes. The responses to the questionnaire refer to the time it takes to reach the OR for an ambulatory person (e.g., obstetrician), rather than the duration of the patient’s transfer. However, these responses, indicate that most respondents are confident in their ability to quickly transfer the woman to the OR to perform an emergency CS. Another important limitation is the lack of data on the safety of attempted vaginal birth, and its relationship to organizational factors, as information on uterine rupture and asphyxia is not routinely recorded in the national registries. A final limitation is that the TOLAC rate is an overestimate of the true TOLAC rate, which is based on the sum of the number of VBACs and the number of unplanned repeat CS. Planned mode of delivery is not recorded in perinatal registries.

When discussing the mode of delivery with a woman with a history of CS, the obstetrician may be reluctant to offer a TOLAC if the labor and deliveryunit has less capacity to manage an emergency CS. Among the organizational differences, we chose to collect information and analyze factors that are likely to influence the decision-to-delivery interval. There is no consensus on the best decision-to-delivery interval, but it should be as short as possible in the event of complications during TOLAC [12–15, 19, 20]. This is the primary concern when offering a TOLAC, both for patient safety and from a medicolegal perspective. If uterine rupture is suspected, it is recommended to perform an emergency CS and deliver the baby within 30 min of the decision, which is not easy to achieve [12, 19]. Previous studies have shown that the transfer time to the OR and the lack of medical staff on site are the most important delaying factors [21–24].

A significant proportion of maternity units in Belgium have fewer than 1500 deliveries per year. In smaller units, it is more difficult to provide a permanent medical presence on site (especially the anesthesiologist and the obstetrician) and a dedicated OR in the delivery room. This calls into question the safety of TOLAC in smaller units, because of the risk of perinatal asphyxia and poor neonatal outcome in the event of uterine rupture. The recommendation in these situations should not be to perform more ERCS in women with a history of CS, but to have a medical presence on site when a woman with a CS scar is in labor and to alert the OR of a possible urgent CS. If these options are not realistic, referring patients with a CS scar and a desire for TOLAC to a larger unit could be an alternative strategy.

We found a statistically significant association between the presence of 24-h on-site medical staff, which is an important determinant of the ability to perform an emergency CS, and the mean proportion of TOLAC [25]. Previous studies have suggested that higher TOLAC and Vaginal Birth After C-section (VBAC) rates were associated with 24/7 on-site anesthesiologist presence, which was more likely in larger volume hospitals [26, 27]. A single-center study showed significantly higher TOLAC and VBAC rates when women were cared for by physicians ona night float call schedule compared with physicians on a ‘traditional’ schedule. Night float call was defined as a schedule in which the provider had clinical responsibility only for a day or night shift, with no other clinical responsibilities before or after the period of responsibility for laboring women [25].

The independent effect of the other organizational factors could not be assessed in a multivariable model, because of significant co-linearity between them (i.e., larger hospitals have a NICU, a readily accessible OR and on site medical staff).

We found no correlation between the provision of TOLAC and its success (VBAC), which may be partly explained by the overestimation of TOLAC. If anything, there was a small trend toward higher success with a higher proportion of TOLAC. There was a significant negative correlation between the overall proportion of CS and the proportion of TOLAC and in case of TOLAC, VBAC. This correlation was also reported at the country level in an international study of uterine rupture [16]. Countries with lower CS rates had higher rates of TOLAC and VBAC. We hypothesize that these associations are also present at the individual level. The TOLAC and VBAC rates may vary among obstetricians depending on their willingness to avoid CS, the type of obstetric practice and the case mix of the maternity unit. The level of involvement of midwives may also differ between maternity units and influence TOLAC rates [28, 29]. The difference in TOLAC rates between the northern and southern parts of Belgium could be partly explained by this organizational factor which should be investigated in future studies of TOLAC provision.

## Conclusion

Some organizational differences appear to affect the provision of TOLAC to women with a history of CS. Addressing these organizational factors, however, may not be sufficient to change practice, as the general tendency to perform a CS in the maternity unit is the main contributor to the percentage of TOLAC.

### Supplementary Information

**Additional file 1:** **Additional Table 1.** Proportion of VBAC in case of TOLAC, according to location, size and organization of the maternity. **Additional Table 2.** Overall proportion of CS, according to location, size and organization of the maternity.

## Data Availability

The datasets used and analyzed during the current study are available from the corresponding author on reasonable request.

## Abbreviations

c-section: Cesarean section
DDI: Decision-to-delivery interval
NICU: Neonatal Intensive Care Unit
OR: Operating room
TOLAC: Trial of Labor After Cesarean
VBAC: Vaginal Birth after Cesarean
