# Supplementary material for: Influence of organizational factors on the offer and success rate of a trial of labor after cesarean section in Belgium: an ecological study
Source: BMC Pregnancy Childbirth. 2023 Sep 22;23:684. doi: 10.1186/s12884-023-05984-w (PMC10515028; doi:10.1186/s12884-023-05984-w)
Supplement: Supplementary file 1 — Additional file 1: Additional Table 1. Proportion of VBAC in case of TOLAC, according to location, size and organization of the maternity. Additional Table 2. Overall proportion of CS, according to location, size and organization of the maternity. [file 12884_2023_5984_MOESM1_ESM.docx]

Additional Table 1: Proportion of VBAC in case of TOLAC, according to location, size and organization of the maternity.

|  | Proportion of VBAC | |
| --- | --- | --- |
| Characteristic (number of maternities) | Mean (SD) | Range and p-value |
| Location |  | p=0.13 |
| Flanders (58) | 67.8% (7.4) | 52% to 83% |
| Brussels-Wallonia (37) | 65.4% (7.7) | 36% to 80% |
| Number of deliveries/years |  | p=0.75 |
| >1500 (22) | 67.2% (5.7) | 58% to 80% |
| ≤1500 (73) | 66.7% (8.1) | 36% to 83% |
| Gynecologist on-site 24/7 |  | p=0.85 |
| Yes (24) | 66.6% (5.9) | 57% to 80% |
| No (71) | 66.9% (8.1) | 36% to 83% |
| Anesthetist on-site 24/7 |  | p=0.14 |
| Yes (52) | 67.9% (6.4) | 54% to 83% |
| No (43) | 65.6% (8.3) | 36% to 80% |
| Pediatrician on-site 24/7 |  | p=0.70 |
| Yes (27) | 67.3% (6.3) | 51% to 80% |
| No (68) | 66.7% (8.0) | 36% to 83% |
| Both gynecologist & anesthetist on-site |  | p=0.47 |
| Yes (20) | 67.7% (5.6) | 60% to 80% |
| No (75) | 66.6% (8.0) | 36% to 83% |
| Presence of NICU |  | p=0.86 |
| Yes (19) | 67.1% (5.7) | 60% to 80% |
| No (76) | 66.8% (8.0) | 36% to 83% |
| Location of the operating room (OR) |  | p=0.99 |
| In the delivery room (24) | 66.9% (6.4) | 57% to 80% |
| On the same floor (21) | 66.6% (7.8) | 55% to 78% |
| On a different floor (50) | 66.9% (8.1) | 36% to 83% |
| Reported transfer time to the OR |  | p=0.17 |
| 1 minute or less (31) | 68.5% (6.7) | 55% to 80% |
| 2 to 5 minutes (57) | 66.2% (8.2) | 36% to 83% |
| More than 5 minutes (7) | 64.8% (4.9) | 58% to 72% |

Additional Table 2: Overall proportion of CS, according to location, size and organization of the maternity.

|  | Proportion of CS | |
| --- | --- | --- |
| Characteristic (number of maternities) | Mean (SD) | Range and p-value |
| Location |  | p=0.54 |
| Flanders (58) | 20.9% (3.3) | 15% to 31% |
| Brussels-Wallonia (37) | 21.3% (4.0) | 14% to 30% |
| Number of deliveries/years |  | p=0.31 |
| >1500 (22) | 20.4% (3.0) | 16% to 27% |
| ≤1500 (73) | 21.2% (3.7) | 14% to 31% |
| Gynecologist on-site 24/7 |  | p=0.70 |
| Yes (24) | 21.3% (3.8) | 16% to 31% |
| No (71) | 21.0% (3.5) | 14% to 30% |
| Anesthetist on-site 24/7 |  | p=0.63 |
| Yes (52) | 20.9% (3.1) | 15% to 31% |
| No (43) | 21.2% (3.7) | 14% to 30% |
| Pediatrician on-site 24/7 |  | p=0.28 |
| Yes (27) | 21.7% (3.7) | 16% to 30% |
| No (68) | 20.8% (3.5) | 14% to 30% |
| Both gynecologist & anesthetist on-site |  | p=0.54 |
| Yes (20) | 21.5% (4.0) | 16% to 31% |
| No (75) | 20.9% (3.4) | 14% to 30% |
| Presence of NICU |  | p=0.17 |
| Yes (19) | 22.1% (3.8) | 16% to 31% |
| No (76) | 20.8% (3.5) | 14% to 30% |
| Location of the operating room (OR) |  | p=0.71 |
| In the delivery room (24) | 21.5% (3.6) | 16% to 31% |
| On the same floor (21) | 21.1% (4.4) | 16% to 31% |
| On a different floor (50) | 20.8% (3.2) | 14% to 30% |
| Reported transfer time to the OR |  | p=0.19 |
| 1 minute or less (31) | 20.7% (3.8) | 16% to 31% |
| 2 to 5 minutes (57) | 20.9% (3.3) | 14% to 30% |
| More than 5 minutes (7) | 23.4% (4.2) | 20% to 30% |
